# Genome-resolved metaproteomic characterization of preterm infant gut microbiota development reveals species-specific metabolic shifts and variabilities during early life

**DOI:** 10.1186/s40168-017-0290-6

**Published:** 2017-07-10

**Authors:** Weili Xiong, Christopher T. Brown, Michael J. Morowitz, Jillian F. Banfield, Robert L. Hettich

**Affiliations:** 1 Biosciences Division, Oak Ridge National Laboratory, Oak Ridge, TN USA; 2 Department of Earth and Planetary Science, University of California, Berkeley, CA USA; 3 School of Medicine, University of Pittsburgh, Pittsburgh, PA USA; 4 Chemical Sciences Division, Oak Ridge National Laboratory, Bethel Valley Rd, Oak Ridge, TN 37831 USA

**Keywords:** Metaproteomics, Human infant gut, Shotgun proteomics, Genome resolved, Microbial metabolic functions

## Abstract

**Background:**

Establishment of the human gut microbiota begins at birth. This early-life microbiota development can impact host physiology during infancy and even across an entire life span. However, the functional stability and population structure of the gut microbiota during initial colonization remain poorly understood. Metaproteomics is an emerging technology for the large-scale characterization of metabolic functions in complex microbial communities (gut microbiota).

**Results:**

We applied a metagenome-informed metaproteomic approach to study the temporal and inter-individual differences of metabolic functions during microbial colonization of preterm human infants’ gut. By analyzing 30 individual fecal samples, we identified up to 12,568 protein groups for each of four infants, including both human and microbial proteins. With genome-resolved matched metagenomics, proteins were confidently identified at the species/strain level. The maximum percentage of the proteome detected for the abundant organisms was ~45%. A time-dependent increase in the relative abundance of microbial versus human proteins suggested increasing microbial colonization during the first few weeks of early life. We observed remarkable variations and temporal shifts in the relative protein abundances of each organism in these preterm gut communities. Given the dissimilarity of the communities, only 81 microbial EggNOG orthologous groups and 57 human proteins were observed across all samples. These conserved microbial proteins were involved in carbohydrate, energy, amino acid and nucleotide metabolism while conserved human proteins were related to immune response and mucosal maturation. We identified seven proteome clusters for the communities and showed infant gut proteome profiles were unstable across time and not individual-specific. Applying a gut-specific metabolic module (GMM) analysis, we found that gut communities varied primarily in the contribution of nutrient (carbohydrates, lipids, and amino acids) utilization and short-chain fatty acid production.

**Conclusions:**

Overall, this study reports species-specific proteome profiles and metabolic functions of human gut microbiota during early colonization. In particular, our work contributes to reveal microbiota-associated shifts and variations in the metabolism of three major nutrient sources and short-chain fatty acid during colonization of preterm infant gut.

**Electronic supplementary material:**

The online version of this article (doi:10.1186/s40168-017-0290-6) contains supplementary material, which is available to authorized users.

## Background

Microbes colonize most internal and external surfaces of the human body and influence many aspects of human physiology. The largest microbial community is found in the human gastrointestinal tract (“gut”), which is composed of up to 56 trillion microbial cells [1], comprising thousands of different species and five million unique genes [2]. Microbes residing in the gut interact with each other and the host; play important roles in host nutrient availability through the production of vitamins, short-chain fatty acids (SCFA), and amino acids; train the immune system to tolerate commensal bacteria; protect against pathogens; and contribute to intestinal epithelium maturation and integrity [3]. Dysbiosis of the gut microbiota has been linked to many diseases, such as Crohn’s disease [4–6], diabetes [7, 8], and autoimmune diseases [9]. The establishment of gut microbiota begins during infancy, and emerging evidence suggests that this initial colonization has lifelong effects on human health [10]. A rapidly increasing number of studies have focused on understanding the establishment of the microbiota at birth, or microbiota associations with infant health and disease.

Although originally thought to be born sterile, the presence of microbes in placental and meconium samples has suggested that infants may be colonized by small populations of microbes prior to birth [11]. Regardless, newborn infants are exposed to large numbers of bacteria from the mother and the environment at birth. Typically, the initial colonizers of the gut are facultative anaerobes and within days or weeks there is a shift from facultative to obligate anaerobes [12, 13]. The establishment of the microbiota is influenced by multiple factors, including gestational age, delivery mode, birth weight, diet and exposure to antibiotics [14–16]. For example, the microbiota of infants born vaginally resembles the mother’s vaginal and fecal microbiota, whereas the microbiota of infants born by cesarean section is more similar to the microbiota of skin or other environments [15]. It has also been suggested that C-section-delivered infants have lower diversity gut microbial communities compared to vaginally delivered infants [17]. The infant gut undergoes a rapid increase in the abundance and diversity of microbial communities during the first few weeks [18]. After 2–3 years of life, the gut microbiota become more stable and adult-like [18]. Large variations have been observed among different individuals and also over time within the same individual [19]. It remains to be determined what and how specific factors (e.g. host genetics, environment, diet, and/or interplay between host and microbiota) determine the path of microbiota development, and how different paths relate to health and disease status. This is particularly critical for premature infants who may have a delayed and aberrant microbiota.

Infants born prematurely are at higher morbidity and mortality risk due to immature organ systems that are not properly adapted to extrauterine life [20]. These infants are susceptible to inflammatory disorders as a result of their poorly developed immune system and prenatal/postnatal events that inappropriately modulate immunity (e.g. perinatal infection and inflammation) [14, 21]. Among premature infants, the incidence of sepsis and necrotizing enterocolitis (NEC) have remained high and have been associated with aberrant gut microbial colonization during first few weeks of life [22, 23]. The role of bacterial colonization in neonatal NEC has been suggested by a number of observations, including the identification of pneumatosis intestinalis (gas in the bowel wall), which is most likely produced by intestinal bacteria, occurrence of outbreaks in hospital, and resolution of inflammation after treatment with antibiotics [24–26]. Recently, Raveh-Sadka et al. analyzed gut communities in a large number of premature infants during a cluster of NEC infections. Results showed that gut colonization was largely unique among infants and that no single bacterial strain was shared among all infants who developed NEC [27]. This suggests that the disease is not caused by a single bacterial strain, but rather may be associated with multiple deleterious bacteria that disrupt essential activities of mutualistic microbes. Characterization of functional activities and temporal profiles of the gut microbiota during early colonization may further enhance our understanding of the role of gut microbiota in the onset of NEC.

Mass spectrometry-based metaproteomics has been widely used to characterize the proteome of microbial communities and has emerged as a valuable tool in investigating the gut microbiota [12, 28, 29]. In particular, coupling genome-resolved community metagenomics with proteomics allows us not only to explore functional roles of microbial communities in the gut ecosystem, but also to link specific metabolic functions to microbial community members. We have recently described an enhanced metaproteomic approach for the microbiome characterization of infant fecal samples [30]. Here, we employed the approach and expanded our analysis to a total of 30 fecal samples collected during the first 3 months of life of four premature infants, one of which developed NEC. By integrating metaproteomics with metagenomics, we identified temporal and inter-individual variations in community protein abundance, determined conserved metabolic functions of both human host and gut microbiota, and revealed functional differences of gut communities in the metabolism of nutrients and short-chain fatty acids during microbial colonization of the premature infant gut.

## Results and discussion

### Samples and metaproteomic measurements

**Fig. 1:**
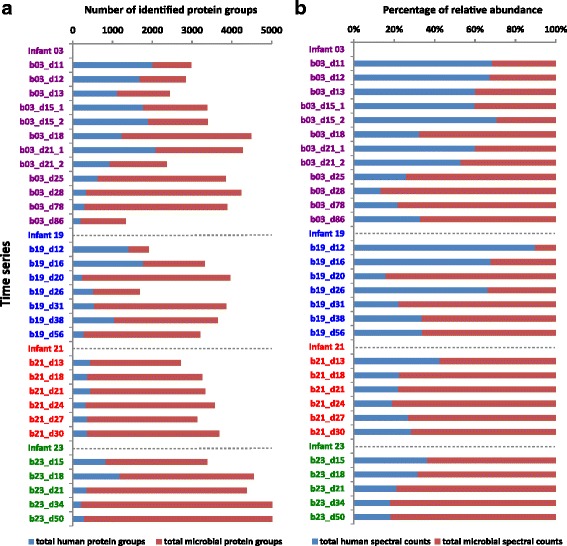
Number of human and microbial protein groups identified (**a**) and relative abundance of human/microbial spectra (**b**) over time. Spectral counts are normalized by the number of total collected spectra and averaged between duplicate runs

Thirty fecal metaproteomes from four preterm infants (03, 19, 21, and 23) collected over the first 3 months after birth were examined by a shotgun metaproteomic approach (each sample was measured in technical duplicate). One of the four infants (infant 19) developed a case of sepsis and another infant (infant 21) developed necrotizing enterocolitis (NEC) (see Materials and Methods and Additional file 1 for more details). These samples were collected as part of a prior genome-resolved metagenomics study (Raveh-Sadka et al*.*) [31], and metaproteomic measurement resulted in an average of 108,763 spectra and 17,754 peptides per sample. To alleviate the ambiguity associated with shared peptides, identified proteins were clustered into protein groups (see Materials and Methods for more details). Human and microbial proteins were both monitored, providing a total of 12,568 (9318 microbial and 3250 human), 9665 (7397 microbial and 2268 human), 7091 (6349 microbial and 742 human), and 11,649 (10,330 microbial and 1319 human) protein groups across all time points for infants 03, 19, 21, and 23, respectively (Additional file 2 and Fig. 1). In general, the measuring depth of human proteins decreased with time as microbial proteins became more dominant and abundant, with approximately 200 human protein groups being identified at later time points. Despite the overall trend towards decreased representation of human proteins, variations were observed over time. For example, dramatic decreases of microbial proteins from the second sample collected on day of life (DOL) 21 (21_2) of infant 03, and DOL 26 of infant 19. Interestingly, the decrease observed at DOL 26 of infant 19 coincided with antibiotics use, suggesting that the treatment effectively suppressed the microbiota. However, the decrease noticed at DOL 21_2 of infant 03 might be related to an unmatched database, as a metagenome was only available for the first sample collected on DOL 21. Major microbiota changes occurring between samples would make our constructed database less relevant to the second sample, which could have led to fewer microbial protein identifications.

### Varying metaproteome coverage of studied infant gut communities

**Fig. 2:**
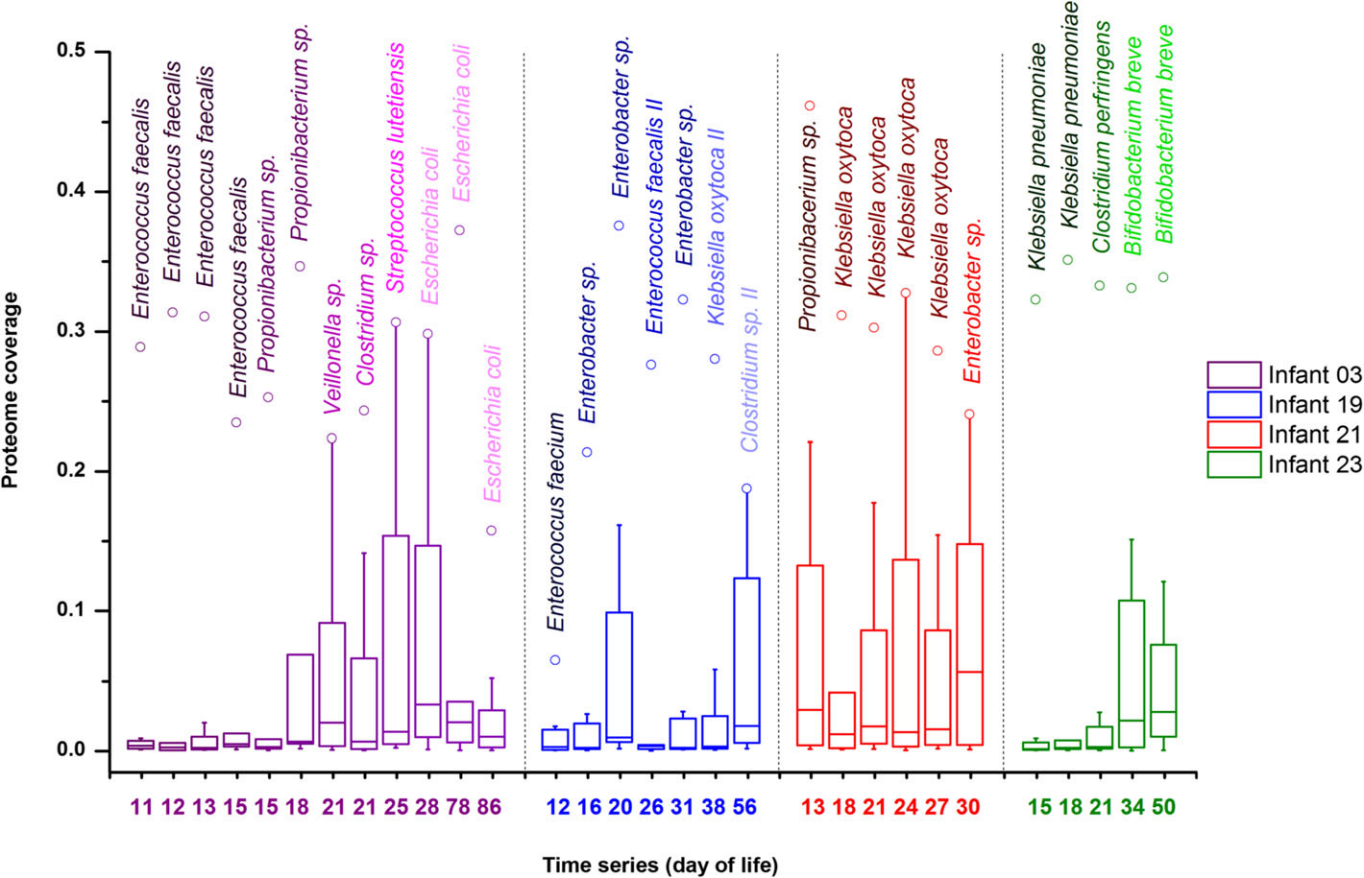
Boxplot comparing organism-specific proteome coverage across samples. Day of life (DOL) of each infant is shown on the *x*-axis. For each sample, the organism having the highest proteome coverage is listed and represented as *open circles*. Only near-complete assembled genomes are included

One of the most important considerations for metaproteomic experiments is the biodiversity and the inherent biological dynamic range within the environment being analyzed. The number of organisms present and their relative abundance directly affect proteome coverage. A typical 24-h LC-MS/MS experiment identifies a few thousand proteins regardless of sample complexity, due to the constrained dynamic range and duty cycle of the mass spectrometer. Therefore, more complex communities yield lower average proteome coverage per organism, and species with higher abundance or activity tend to have a larger percentage of the proteome that can be detected. By integrating strain-resolved metagenomics with deep metaproteomic measurements, we were able to characterize organism-specific proteome coverage across time, as shown in Additional file 3. Since not all predicted proteins are expressed under one condition and the genome size varies among organisms, a typical proteome analysis of a single microbial isolate can identify approximately 50 to 80% of the predicted proteins [32, 33]. Preterm infant gut microbial communities harbor less diversity than other more complex communities (e.g. human adult gut and soil microbiota) and generally contain a limited number of highly abundant organisms. Here, in total, up to 45% (e.g. *Propionibacterium sp.* in DOL 13 of infant 21) of the predicted proteins for an individual organism could be measured and identified. The distribution of different proteome coverage for species/strains in each sample was displayed in Fig. 2, with the species/strain having the highest proteome coverage listed. During early time points, the gut communities were dominated by one or two species (i.e., the outlier with the highest proteome coverage, which is represented as an open circle), and thus proteomes of most other species had low coverage (represented as tight box width). Advancing DOL correlated with a broader distribution of proteome coverage across organisms, indicating that a greater range of bacteria became abundant/active during the colonization process. However, we noticed that distributions of proteome coverage for communities from infant 21 were less skewed and variable as compared to other infants, possibly due to the lower species richness in the gut microbiome of this infant.

### Temporal shifts in microbial activity

**Fig. 3:**
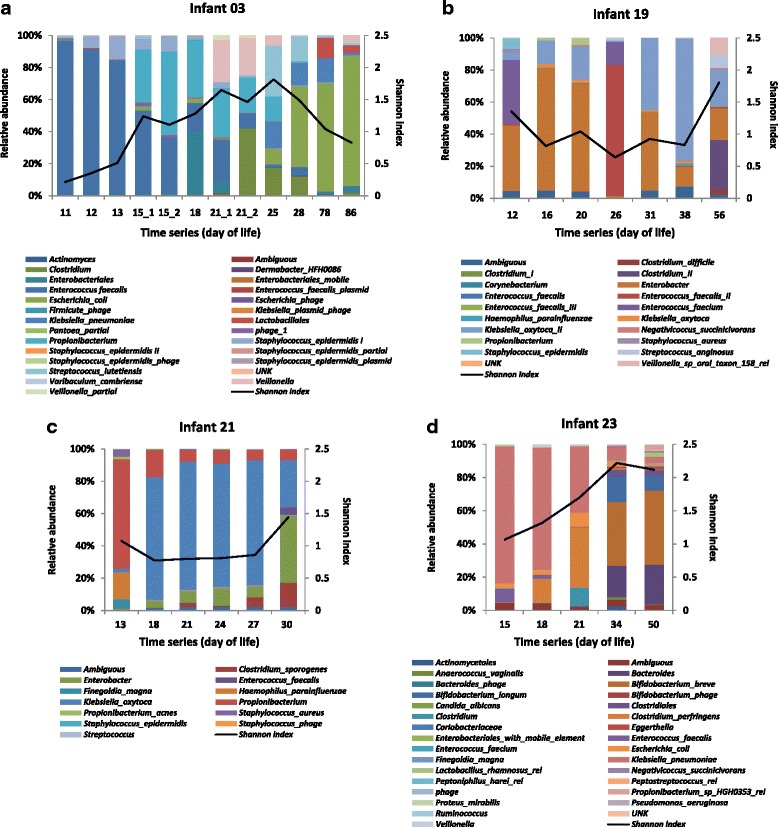
Taxonomic shifts in infants **a** 03, **b** 19, **c** 21, and **d** 23 based on protein abundance. Relative abundance of microbial community is based on assigning proteomic data to constructed metagenomes. Shannon diversity index is used to characterize species diversity (both abundance and evenness of the species present) and calculated from the relative abundance of proteins at the species level

To further investigate the activity of microbial community members, the proteomic data was matched to genomes previously reconstructed for microbial community members by Raveh-Sadka et al*.* [31] (Fig. 3). The vast majority of proteins (>99%) were unambiguously assigned to a specific species/strain, but proteins belonging to closely related strains (e.g. *Klebsiella oxytoca* and *Klebsiella oxytoca II* in infant 19; *Bifidobacterium longum* and *Bifidobacterium breve* in infant 23), or identical proteins from different species were indistinguishable. Proteins were identified from 25, 18, 12, and 29 different species/strains for infants 03, 19, 21, and 23 respectively, showing that microbiomes of infant 19 and 21 were less complex. We resolved proteins from several phages, identifying a few structural proteins and a majority of unknown-function proteins. In addition, a low abundance of proteins from *Candida albicans* were identified in infant 23. Microbial composition was largely different between infants, and *Enterococcus faecalis* was the only species that was shared by all infants. However, a number of species were common between the twins 19 and 21, such as *Staphyloccoccus aureus*, *Enterobacter cloacae*, *Klebsiella oxytoca*, and *Haemophilus parainfluenzae*, suggesting that the gut microbiome may be impacted by host genetics or exposure to the same environment. The environmental impact might be less relevant because infant 23 was co-hospitalized with the twin infants but was not colonized by the abovementioned species. In addition to the remarkable inter-individual variation, the relative protein abundance and species diversity (Shannon index) of the microbial community within each infant also varied dramatically during the early colonization phase. Intriguingly, apparent differences were observed between the genomic and proteomic patterns on certain DOLs (genomic results have been shown in a prior study [31]), suggesting a few species that are more active in spite of low abundance, such as *Streptococcus lutetiensis* in DOL 25 of infant 03, *Clostridium sp.* in DOL 56 of infant 19, and *Propionibacterium sp.* in DOL 13 of infant 21. In this last sample, *Propionibacterium sp.* accounted for almost 70% relative abundance in the proteome measurement while its DNA sequence reads only comprised of 15% of the community. These findings could have significant impacts on our understanding of the balance between microbial population structure and dominant metabolic activities.

Moreover, we analyzed different samples collected on the same DOL (pairs 15_1 and 15_2; 21_1 and 21_2 of infant 03, Fig. 3a), to investigate whether the microbiome is stable within a day. Database searching of samples 15_2 and 21_2 was conducted based on metagenomes from different fecal samples collected on the same DOL (15_1 and 21_1, respectively). For both pairs, distributions of microbial protein abundance showed different patterns between the two samples. On DOL 15, the microbiota composition remained almost the same but the most active species shifted from *Enterococcus faecalis* to *Propionibacterium sp.* in the later fecal sample. However, on DOL 21, a new dominant colonizer—*Clostridium sp.* appeared, which greatly changed the community composition in the second sample. A Pearson correlation of *r* = 0.9 was found between samples on DOL 15 while the correlation between samples on DOL 21 was *r* = 0.53, indicating that the gut microbiome changed greatly within a day, although the cause of the shift was not apparent. The variance was less likely due to a technical issue, as it was observed that *Clostridium sp.* stayed relatively abundant until DOL 25. So it was more likely that *Clostridium sp.* colonized and developed rapidly on late DOL 21, resulting in significant changes in the microbiome. The finding of a shift in community activity is not surprising considering a recent study that also recognized rapid and reproducible alterations of human adult gut microbiome by dietary interventions [34].

### Temporal and individual variabilities of infant gut metaproteome profiles

**Fig. 4:**
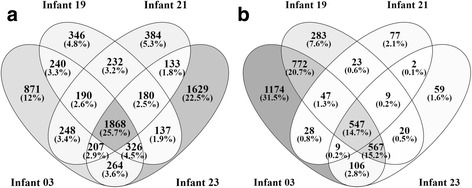
Venn diagram of microbial EggNOGs (**a**) and human protein groups (**b**) in all four infants. A total of 4214, 3519, 3442, and 4744 microbial EggNOGs and 3250, 2268, 742, and 1319 human proteins are identified in infants 03, 19, 21, and 23 respectively

As discussed above, the microbial composition and proportions not only vary dramatically during the early colonization phase but also can be remarkably different among infants, and therefore we employed the strategy of annotating identified proteins by orthologous groups to determine the presence and abundance of different proteins across samples. By using the recently updated EggNOG orthologous database [35], annotations were obtained for over of 85% microbial genes and 98% of identified microbial proteins. In total, 4214, 3519, 3442, and 4744 non-redundant microbial EggNOGs were assigned for metaproteomes of infant 03, 19, 21, and 23 respectively (Fig. 4a). Among all identified microbial EggNOGs, 1868 (26%) were commonly identified in all four infants. The highest number of unique EggNOGs (1629, 22.5%) was found in infant 23. When considering samples collected at multiple time points within an infant, 1249 EggNOGs were found in at least half of the samples, but only 81 were present across all 30 samples (Additional files 4 and 5), showing large dissimilarity of these proteome profiles. The common 81 EggNOGs mainly participate in c*arbohydrate transport* using bacteria-specific phosphotransferase system (PTS), *carbohydrate metabolism* including glycolysis, pentose phosphate pathway and galactose degradation, *energy production* involving ethanol production and glycerol degradation, *amino acid* (e.g. glutamate, arginine and glycine) *metabolism*, *nucleotide metabolism*, *transcription*, *translation* and chaperonin*-*assisted *protein folding*, revealing a conserved functional profile of gut microbiota. In addition, 547 human proteins (15%) were commonly identified in four infants, but only 57 proteins were detected across all samples (Fig. 4b and Additional file 4). Unlike essential microbiome functions which mainly support cell growth and maintenance, these common host proteins in the gut included proteins related to lipid and protein digestion, antibacterial activity, innate immune response, and gut mucosal barrier development and protection (Additional file 5). Notably, as essential components in the infant innate immunity, intestinal barrier and immune factors were present at all times. These factors might constantly fine-tune the activities of the microbial community in order to maintain the homeostatic balance between the developing gut microbiota and the host environment [36].

**Fig. 5:**
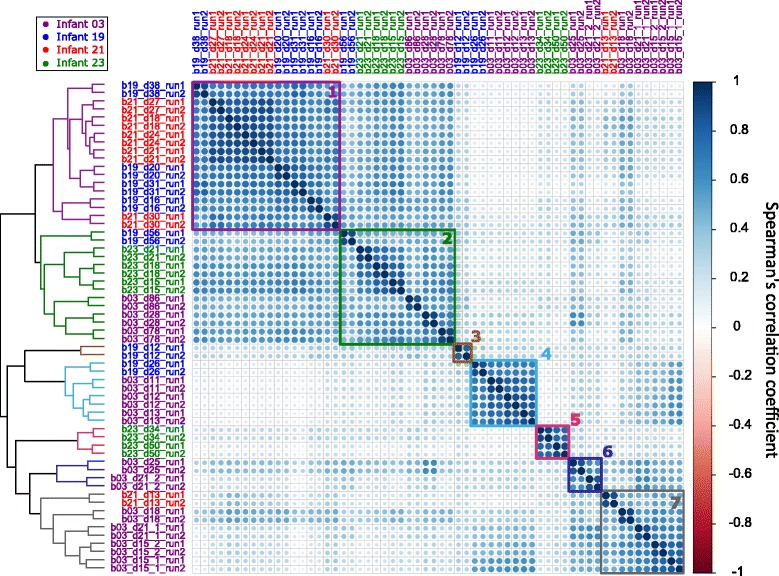
Correlation plot with hierarchical clustering of microbial EggNOGs. Shown is a correlation matrix plot showing the spearman correlation of each mass spectrometric measurement. Sample names are composed of the infant number (*b*), day of life (*d*) and the measurement number (*run*). The color of sample names indicates different infants. Rows are hierarchically clustered using complete linkage clustering and the seven clusters are highlighted by *colored boxes*

To further assess the correlation of studied metaproteome profiles, Spearman’s rank correlations with hierarchical clustering were applied, and seven clusters were identified (Fig. 5). Typically, samples collected from adjacent time points of the same infant were highly correlated, but not all samples clustered by individual. Intriguingly, samples from different infants (DOL 11 of infant 03 and DOL 26 of infant 19) collected after antibiotics treatment clustered together (cluster 4). This might be related to an abundance of *E. faecalis* occurring in both samples after antibiotics use. Potential antibiotic resistance proteins identified in *E. faecalis* were analyzed by CARD [37] and shown in Additional file 6. Additionally, we found that the microbiota seemed to be restored after antibiotic treatment in infant 19, as samples taken before and after antibiotics treatment clustered together (DOL 20 and 31), but not with the sample from the administration period (DOL 26). As mentioned above, a number of bacterial species were shared by twin infants 19 and 21. Microbial proteomes from the two infants were also closely related (cluster 1). A recent study has revealed a subject-specific and stable gut metaproteome in human adults [38]. However, our observations showed that during infancy, the proteome was less individualized and more unstable as compared with that observed in human adults. Infant age, genetic background and antibiotics use are likely to be major determinants for the microbiota development in neonates. Among conserved functional groups identified in adults from the previous study, formate acetyltransferase, glutamate dehydrogenase, and three glycolytic enzymes were also conserved in infants studied in our study. However, enzymes involved in the production of butyrate (butyryl-CoA dehydrogenase) and vitamin B12 (sirohydrochlorin cobaltochelatase) were conserved in human adults but only found in a portion of infant samples, suggesting that the infant intestinal microbiota is variable and has not stabilized for these functions during the early colonization.

In order to further investigate proteins that distinguish metaproteome clusters, proteins with significantly different abundances between clusters were identified (Additional file 7). Notably, cluster 5 was the most distinguishable cluster, having the longest distance from it to other clusters. An abundance of proteins involved in carbohydrate metabolism were identified in cluster 5, for example, ABC transporter proteins (likely for sugars), beta-galactosidase and galactose-1-phosphate uridylyltransferase participating in the degradation of lactose, and xylulose-5-phosphate/fructose-6-phosphate phosphoketolase acting as a key enzyme in the “Bifid shunt” pathway. The “Bifid shunt” in which carbohydrates are fermented via phosphoketolase is a unique process in *Bifidobacteria*. Samples (DOL 34 and 50 of infant 23) clustered in cluster 5 had a dominance of *Bifidobacterium breve* in both communities. Interestingly, this infant 23 is the only infant with dominant obligate anaerobic *Bifidobacterium*. Therefore, cluster 5 was clearly distinct from other metaproteome clusters, possibly due to the distinct carbohydrate utilization of *Bifidobacterium*.

### Metabolic profiles in association with shifts in microbial communities

To assess the diversity and stability of metabolic functions across these communities, a recently developed gut-specific framework was applied to infer species-associated GMMs (gut metabolic modules) for our dataset [39]. A total of 104 GMMs were inferred for all infants, with module coverage higher than 0.556 (55.6%) (the optimal coverage cutoff is inferred during module prediction), and the temporal abundances of all microbial members that participate in the module were determined (Additional file 8).

#### Modules associated with carbohydrate, amino acid, and lipid metabolism

**Fig. 6:**
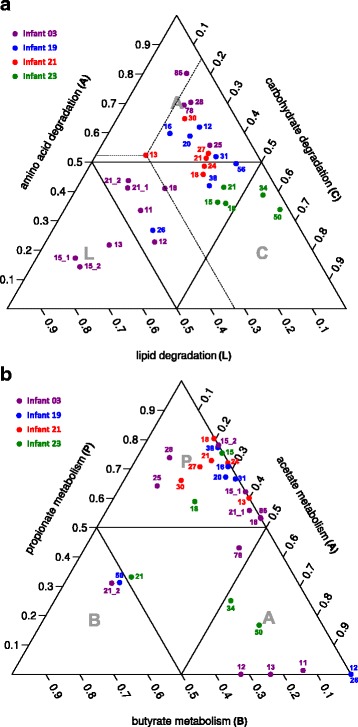
Tri-plot representation of the gut communities in the exploitation of three major energy sources (**a**) and the metabolism of short-chain fatty acids (**b**). For each metabolic category/trait (e.g. carbohydrate/lipid/amino acid degradation and propionate/butyrate/acetate metabolism), the abundance was defined as summed protein abundances of GMMs associated with the category divided by the number of GMMs in that category. Shown tri-plot representation is based on the relative abundance of the three defined categories. The *dashed lines* give an example of the proportions of carbohydrate, lipid and amino acid degradation for the sample DOL13 of infant 21. Each *corner triangle* labeled with one-letter category name (e.g. amino acid (*A*); lipid (*L*); carbohydrate (*C*); propionate (*P*); butyrate (*B*); acetate (*A*)) represents dominant proteomic investment in that particular metabolic category. Day of life (DOL) is shown on each sample/dot

We assessed infant gut communities in the exploitation of three major energy sources (carbohydrate, amino acid, and lipid) across time. All measured communities were found to use all three types of nutrients, but the relative abundance of saccharolytic (carbohydrate degradation), proteolytic (amino acid degradation), and lipolytic (lipid degradation) fermentation modules varied among communities (Fig. 6a, Additional file 8). Among four infants, a larger separation in the utilization of nutrients was identified in infant 03 over time, while samples from the same infant were closely clustered for the other three infants. This is likely due to the longer sampling period covered for infant 03.

Intriguingly, gut communities associated with infant 03 exhibit high levels of lipolytic fermentation at early time points and shifted towards dominant proteolytic fermentation over time. The availability of genome sequences reconstructed from matched metagenomes enabled us to determine the contribution of specific species to these functions. *Enterococcus faecalis* was dominant in the community over DOL 11 to 15 and contributed significant protein abundance to glycerol degradation through expression of glycerol dehydrogenase and dihydroxyacetone kinase. The source of glycerol may have been the complete digestion of breast milk triglycerides via host pancreatic lipase and breast milk-derived bile salt-stimulated lipase [40], which were both identified in the samples. Thereby, free glycerol can be utilized by gut bacteria and converted to the glycolysis intermediate dihydroxyacetone phosphate, potentially for ATP production. On DOL 18, the proportional contribution of bacteria to amino acid degradation began to increase, mainly contributed by *Enterbacteriales sp*. For example, aspartate aminotransferase and aspartate ammonia-lyase were both identified, allowing the conversion of l-aspartate to oxaloacetate and fumarate, respectively. Both products can be further consumed via entering the TCA cycle. Starting from DOL 21, glutamate degradation was observed and became relatively abundant, which was contributed by *Clostridium sp.* and *Escherichia coli*. We identified glutamate decarboxylase A and B in *E. coli*, which were required for the degradation of l-glutamate to 4-aminobutanoate. 4-Aminobutanoate can be further degraded by 4-aminobutyrate aminotransferase and succinate-semialdehyde dehydrogenase (both were also identified in *E. coli*) to TCA intermediate succinate. The action of all above four proteins, known as “GABA shunt,” channels glutamate into the TCA cycle [41]. Increased tryptophan degradation via tryptophanase was also identified in *E. coli* during the time after DOL 25, which can produce indole, pyruvate, and ammonium. While ammonium can be used as a nitrogen source and indole can act as a signal molecule, pyruvate can be redirected into the TCA cycle.

The trend towards increased protein utilization observed in infant 03 was not identified in other infants. Microbes from infants 19 and 21 remained relatively stable in the fermentation of these three substrates (carbohydrates, amino acids, and lipids), except slightly increased carbohydrate degradation observed during late time points in infant 19. This was mostly driven by *Klebsiella oxytoca II* on DOLs 31 and 38, and *Clostridium sp. II* on DOL 56. A high abundance of beta-galactosidase and alpha-galactosidase were identified in *Klebsiella oxytoca II*, hydrolyzing lactose and melibiose into galactose and glucose. While glucose can directly be utilized via glycolysis, galactose requires five enzymes to be converted into the more metabolically versatile D-glucopyranose 6-phosphate: galactose-1-epimerase, galactokinase, galactose-1-phosphate uridylyltransferase, UDP-glucose 4-epimerase, and phospho-glucomutase, which were all detected in *Klebsiella oxytoca II*. All proteins mentioned above except alpha-galactosidase were also identified in *Clostridium sp. II*. Among the four infants, the gut microbiota of infant 23 presented the highest proportion of carbohydrate fermentation proteins, primarily related to lactose, galactose, and melibiose degradation. These functions were mostly contributed by *Klebsiella pneumoniae* on DOLs 15 to 21 and *Bifidobacterium breve* on DOLs 34 and 50.

However, we did not observe clear associations between macronutrient utilization and clinical outcome, mode of delivery, feeding regimen, or gestational age at delivery. It is important to note that the composition of human milk can vary substantially between mothers and between time points [42]. Since each infant in the study received breast milk, it is possible that this factor could have sharply impacted the variability of gut microbial metabolism.

#### Modules associated with short-chain fatty acid (SCFA) metabolism

SCFAs, primarily acetate, propionate, and butyrate are major end-products of human milk oligosaccharide (HMO) fermentation by intestinal microbiota. We further explored acetate, propionate, and butyrate metabolism in all measured samples (Fig. 6b, Additional file 8) and found a majority of communities predominantly invested in the metabolism of propionate.

Two different pathways were inferred for propionate production (Additional file 9): the succinate pathway and the propanediol pathway. The succinate pathway utilizes succinate as a substrate and employs succinyl-CoA synthetase, methylmalonyl-CoA mutase, methylmalonyl-CoA epimerase, and methylmalonyl-CoA carboxytransferase to convert succinate to propionyl-CoA. All these enzymes were identified and mainly found in species of *Propionibacterium* for all infants. The propanediol pathway is characterized by the conversion of propionaldehyde to propionyl-CoA via CoA-dependent propionaldehyde dehydrogenase as a marker enzyme. This pathway also involves lactaldehyde reductase and propanediol dehydratase, responsible for conversion of l-lactaldehyde to propionaldehyde. The propanediol pathway was primarily found in *Klebsiella pneumoniae* of infants 03 and 23 and in *Klebsiella oxytoca* of infants 19 and 21. The production of propionate in these organisms was further supported by the identification of fucose degradation pathway in the same organism. l-fucose is a major component of glycosylated mucin proteins in the intestinal epithelium and oligosaccharides in human milk [43, 44]. l-fucose isomerase, l-fuculokinase, and l-fuculose phosphate aldolase, enzymes needed to degrade l-fucose to l-lactaldehyde, were all detected in above *Klebsiella* genus that were able to further convert l-lactaldehyde to propionate.

Although most communities exhibited dominant propionate metabolism, samples from DOL 21 of infant 03, DOL 56 of infant 19, and DOL 21 of infant 23 showed relatively high levels of butyrate metabolism. Two different pathways are possible for butyrate production in gut bacteria, but the butyrate kinase pathway was the major one observed in these communities, which employed crotonyl-CoA reductase, phosphotransbutyrylase and butyrate kinase to convert crotonoyl-CoA to butyrate (Additional file 9). This pathway was primarily found in *Clostridium* species, including *Clostridium sp.* of infant 03, *Clostridium difficile* and *Clostridium II sp.* of infant 19, *Clostridium sporogenes* of infant 21, and *Clostridium sp.* and *Clostridium perfringens* of infant 23.

Acetate can be produced by converting acetyl-CoA to acetate via phosphate acetyltransferase and acetate kinase. As opposed to propionate and butyrate production that were mainly controlled by a few organisms in these communities, many organisms were able to produce acetate (Additional file 9). We noticed that samples with very low complexity, either collected from early time points (DOL 11, 12, and 13 of infant 03; DOL 12 of infant 19) or after antibiotics treatment (DOL 26 of infant 19), showed dominant acetate metabolism, possibly because microbes that have the capacity to produce butyrate and propionate have not colonized or have been removed during the time and thus acetate production became relatively predominant. Two samples (DOL 34 and 56) from infant 23 also had relatively high abundance of acetate metabolism enzymes, mainly contributed by *Bifidobacterium breve*. In premature infants, high level of fecal acetate has been associated with increased Bifidobacteria [45].

## Conclusions

In this study, we conducted a metagenome-informed metaproteomic analysis of 30 gut communities from four human preterm infants, allowing us to characterize species-specific gut microbial functional variations between infants during the critical first few weeks of life. The use of sample-matched, genome-resolved metagenomics databases enabled us to identify an average of 2606 microbial protein groups with up to 45% proteome coverage obtained for the most dominant species in each community, and further to reconstruct species-specific metabolic functions and pathways. We found that the pattern of community relative protein abundance varied substantially among individual infants and over the time course, but generally the community began with colonization of facultative anaerobes, such as *Enterococcus and Klebsiella*, followed by the emergence of some obligate anaerobes, for example, *Clostridium*, *Bifidobacterium*, and *Bacteroides*. While rapid shifts in the infant gut microbiome were observed, conserved metabolic pathways were identified, largely associated with microbial cell growth and maintenance. The roles of environmental factors in the early life gut microbiota development are still poorly understood. As opposed to human adults, the infant gut exhibited unstable and individual-unspecific gut metaproteomes, possibly as a result of infant gut microbiota ecosystem being immature/underdeveloped and thus susceptible to disruption by environmental factors. Our data showed a few observations where antibiotics altered the gut metaproteome, but future research is needed to describe the effect of antibiotics on the infant gut microbiota. Given different patterns in the gut communities, our results further revealed species-related metabolic shifts and variations of the infant gut microbiota, particularly in the nutrient exploitation and SCFAs metabolism.

## Methods

### Sample collection

Fecal samples were collected from four preterm infants (03, 19, 21, and 23) over the first 3 months after birth. All samples were collected as part of a prospective cohort study of premature infants with and without necrotizing enterocolitis. The four infants in this study represent four of the first infants within the overall cohort to undergo metagenomics sequencing that also had remaining fecal samples with enough biomass to permit proteomic analysis. The specific samples selected for proteomics were selected on the basis of completeness of bacterial genomes assembled by metagenomics (Raveh-Sadka et al*.*) [31] in order to enable genome-resolved proteomic analysis. Infant 03 was a healthy preterm infant. Infants 19 and 21 were two infants from triplets, among which, 19 developed severe sepsis, but not NEC, while 21 developed NEC and died from NEC totalis. Infant 23 was co-hospitalized with infants 19 and 21, who was healthy aside from mild lung disease. Stool samples were collected on day of life (DOL) 11–86 as available. For the infant 03, samples were collected twice on days 15 (15_1 and 15_2) and 21 (21_1 and 21_2). Additional medical details of four infants were described in a prior study [31] and Additional file 1. Subjects were enrolled and samples were collected according to a research protocol approved by The University of Pittsburgh Institutional Review Board (PRO10090089).

### Sample preparation

~0.3 g raw fecal material was prepared by the indirect enrichment method, as previously detailed with modifications [30]. In brief, raw fecal material was passed through a 20-μm vacuum filter followed by centrifugation to enrich microbial cells. Collected microbial cells were lysed by sodium dodecyl sulfate (SDS) with sonication. One milligram of crude protein extract was then precipitated by trichloroacetic acid (TCA) and washed with ice-cold acetone. Pelleted proteins were re-solubilized in 8 M urea and sonically disrupted to fully solubilize the protein pellet. Denatured proteins were reduced with 5 mM dithiothreitol (DTT), cysteines blocked with 20 mM iodoacetamide (IAA) and digested into peptides with sequencing grade trypsin. The digested samples were then adjusted to 200 mM NaCl, 0.1% formic acid (FA), and filtered through a 10-kDa cutoff spin column filter to collect the tryptic peptides.

### 2D LC-MS/MS measurement

For each sample, obtained peptide samples (50 μg) were analyzed via 22-h MudPIT two-dimensional (2D) nanospray LC-MS/MS system on LTQ-Orbitrap Elite (ThermoFisher Scientific, San Jose, CA). As previously described [46], peptides were separated/eluted in 11 steps (each lasting ~2 h) with an increasing amount of salts (ammonium acetate) followed by organic gradients in each step. Mass spectra were acquired in data-dependent mode: full scans were acquired at 30-k resolution (1 microscan) in the Orbitrap, followed by CID fragmentation of the 20 most abundant ions (1 microscan). Monoisotopic precursor selection was enabled. Unassigned charge and charge state +1 were rejected. Dynamic exclusion was enabled with a mass exclusion width 10 ppm and exclusion duration 30 s. Technical replicates (duplicates) were performed for each sample.

### Peptide and protein identification

Protein databases were constructed for each individual infant by combining proteins predicted from sequenced metagenome (see Raveh-Sadka et al. [31] for more details) collected on multiple days, human protein sequences (NCBI RefSeq_2011), and common contaminants. All MS/MS spectra were searched with the Myrimatch version 2.1 algorithm against the constructed protein database and filtered with IDPicker. Peptide modifications including a static cysteine modification (+57.02 Da), an N-terminal dynamic carbamylation modification (+43.00 Da), and a dynamic oxidation modification (+15.99) were included in all searches. A decoy database consisting of reverse protein sequences was appended to the target database to calculate false discovery rates (FDR). Peptide identifications were filtered by maintaining at least two distinct peptides per protein and a 2% peptide spectrum match-level FDR to achieve confident peptide identifications (FDR <1%). For protein inference, proteins were grouped base on 90% amino acid sequence identity for human proteins and 100% identity for microbial proteins, as previously described [47]. Spectral counts were balanced between shared proteins, and normalized by total numbers of all collected MS/MS in each run.

### Data analysis

EggNOG annotations were obtained using EggNOG database v4.5 via eggNOG-mapper with HMM search mode [35]. Venn diagram was generated using an online tool VENNY 2.1 (http://bioinfogp.cnb.csic.es/tools/venny/index.html). The EdgeR package [48] was used to calculate significantly different protein abundances between clusters via quasi-likelihood negative binomial generalized log-linear model (glmQLFTest). The dataset was normalized based on scaling factors for library sizes, which were determined using a trimmed mean of M values (TMM) between samples. The correlation plot was built using the corrplot package after sorting based on hierarchical clustering. KEGG Orthology (KO) annotations for each protein sequence were assigned by KEGG Automatic Annotation Server (KASS) using GHOSTX search and the SBH (single-directional best hit) method [49]. Gut metabolic modules (GMMs) and tri-plot representations were inferred and visualized from an online tool GOmixer, specifically for gut meta-omics data analysis [39, 50].

## Additional files

Additional file 1: Medical information of infants. (XLSX 9 kb)

Additional file 2: Protein groups and spectral counts identified in all metaproteomic measurements. (XLSX 4908 kb)

Additional file 3: Organism-specific proteome coverage across time series of (a) infant 03, (b) infant 19, (c) infant 21, and (d) infant 23. The percentage of proteome identified is calculated for each organism by assigning peptides to proteins predicted from metagenomics data. Sample with day of life (DOL) is shown on the *x*-axis, and “total” column includes proteins identified across time series. (PDF 39 kb)

Additional file 4: Frequencies of microbial EggNOGs and human protein groups identified across all samples. 81 microbial EggNOGs and 57 human protein groups are identified in all 30 samples. (PDF 92 kb)

Additional file 5: Conserved EggNOGs and human protein groups determined in studied gut metaproteomes. (XLSX 17 kb)

Additional file 6: Potential antibiotic resistance proteins in *E. faecalis* analyzed by CARD. (XLSX 26 kb)

Additional file 7: Proteins associated with metaproteome clusters. Shown are top 25 most significantly different protein groups between each metaproteome cluster pair comparison with p-value less than 0.01. Metaproteome clusters are indicated by colored boxes on top of the heatmap. Sample names at the bottom are composed of the infant number (b), day of life (d) and the measurement number (run). (PDF 2441 kb)

Additional file 8: Gut metabolic modules (GMMs) and abundance inferred from studied gut communities. (XLSX 100 kb)

Additional file 9: Metabolic pathways for propionate, butyrate and acetate formation by representative bacterial species/strains in human preterm infant gut. Species/strains with all enzymes (shown by EC number) in the pathway identified are listed below. The color indicates in which infant the species/strain is identified. (6.2.1.5: succinyl-CoA sythetase; 5.4.99.2: methylmalonyl-CoA mutase; 5.1.99.1: methylmalonyl-CoA epimerase; 2.1.3.1: methylmalonyl-CoA carboxyltransferase; 1.1.1.77: lactaldehyde reductase; 4.2.1.28: propanediol dehydratase; 1.2.1.87: propionaldehyde dehydrogenase; 2.3.1.222: phosphate propanoyltransferase; 2.7.2.15: propionate kinase; 2.3.1.9: acetyl-CoA C-acetyltransferase; 1.1.1.157: 3-hydroxybutyryl-CoA dehydrogenase; 4.2.1.17: enoyl-CoA hydratase; 1.3.8.1: crotonyl-CoA reductase; 2.3.1.19: phosphotransbutyrylase; 2.7.2.7: butyrate kinase; 2.3.1.8: phosphate acetyltransferase; 2.7.2.1: acetate kinase). (PDF 44 kb)

## Abbreviations

CARD: The Comprehensive Antibiotic Resistance Database
DOL: Day of life
DTT: Dithiothreitol
FDR: False discovery rate
GMM: Gut-specific metabolic module
HMOs: Human milk oligosaccharides
IAA: Iodoacetamide
KEGG: Kyoto Encyclopedia of Genes and Genomes
KO: KEGG orthology group
NEC: Necrotizing enterocolitis
PTS: Phosphotransferase system
SCFA: Short-chain fatty acid
SDS: Sodium dodecyl sulfate
TCA: Trichloroacetic acid

## References

[1] Sender R, Fuchs S, Milo R (2016). Revised estimates for the number of human and bacteria cells in the body. PLoS Biol.

[2] Turnbaugh PJ, Ley RE, Hamady M, Fraser-Liggett CM, Knight R, Gordon JI (2007). The human microbiome project. Nature.

[3] Walter J, Ley R (2011). The human gut microbiome: ecology and recent evolutionary changes. Annu Rev Microbiol.

[4] Hall LJ, Walshaw J, Watson AJ (2014). Gut microbiome in new-onset Crohn’s disease. Gastroenterology.

[5] Hofer U (2014). Microbiome: bacterial imbalance in Crohn’s disease. Nat Rev Microbiol.

[6] Wright EK, Kamm MA, Teo SM, Inouye M, Wagner J, Kirkwood CD (2015). Recent advances in characterizing the gastrointestinal microbiome in Crohn’s disease: a systematic review. Inflamm Bowel Dis.

[7] Qin JJ, Li YR, Cai ZM, Li SH, Zhu JF, Zhang F, Liang SS, Zhang WW, Guan YL, Shen DQ (2012). A metagenome-wide association study of gut microbiota in type 2 diabetes. Nature.

[8] Upadhyaya S, Banerjee G (2015). Type 2 diabetes and gut microbiome: at the intersection of known and unknown. Gut Microbes.

[9] Giongo A, Gano KA, Crabb DB, Mukherjee N, Novelo LL, Casella G, Drew JC, Ilonen J, Knip M, Hyoty H (2011). Toward defining the autoimmune microbiome for type 1 diabetes. ISME J.

[10] Matamoros S, Gras-Leguen C, Le Vacon F, Potel G, de La Cochetiere MF (2013). Development of intestinal microbiota in infants and its impact on health. Trends Microbiol.

[11] Tamburini S, Shen N, Wu HC, Clemente JC (2016). The microbiome in early life: implications for health outcomes. Nat Med.

[12] Brooks B, Mueller RS, Young JC, Morowitz MJ, Hettich RL, Banfield JF (2015). Strain-resolved microbial community proteomics reveals simultaneous aerobic and anaerobic function during gastrointestinal tract colonization of a preterm infant. Front Microbiol.

[13] La Rosa PS, Warner BB, Zhou Y, Weinstock GM, Sodergren E, Hall-Moore CM, Stevens HJ, Bennett WE, Shaikh N, Linneman LA (2014). Patterned progression of bacterial populations in the premature infant gut. Proc Natl Acad Sci U S A.

[14] Groer MW, Luciano AA, Dishaw LJ, Ashmeade TL, Miller E, Gilbert JA (2014). Development of the preterm infant gut microbiome: a research priority. Microbiome.

[15] Dominguez-Bello MG, Costello EK, Contreras M, Magris M, Hidalgo G, Fierer N, Knight R (2010). Delivery mode shapes the acquisition and structure of the initial microbiota across multiple body habitats in newborns. Proc Natl Acad Sci U S A.

[16] Guaraldi F, Salvatori G (2012). Effect of breast and formula feeding on gut microbiota shaping in newborns. Front Cell Infect Microbiol.

[17] Song SJ, Dominguez-Bello MG, Knight R (2013). How delivery mode and feeding can shape the bacterial community in the infant gut. Can Med Assoc J.

[18] Koenig JE, Spor A, Scalfone N, Fricker AD, Stombaugh J, Knight R, Angenent LT, Ley RE (2011). Succession of microbial consortia in the developing infant gut microbiome. Proc Natl Acad Sci U S A.

[19] Backhed F, Roswall J, Peng Y, Feng Q, Jia H, Kovatcheva-Datchary P, Li Y, Xia Y, Xie H, Zhong H (2015). Dynamics and stabilization of the human gut microbiome during the first year of life. Cell Host Microbe.

[20] Institute of Medicine. Preterm Birth: Causes, Consequences, and Prevention. Washington, DC: The National Academies Press; 2007;313–14.

[21] Melville JM, Moss TJ (2013). The immune consequences of preterm birth. Front Neurosci.

[22] Stewart CJ, Marrs EC, Nelson A, Lanyon C, Perry JD, Embleton ND, Cummings SP, Berrington JE (2013). Development of the preterm gut microbiome in twins at risk of necrotising enterocolitis and sepsis. PLoS One.

[23] Hunter CJ, Upperman JS, Ford HR, Camerini V (2008). Understanding the susceptibility of the premature infant to necrotizing enterocolitis (NEC). Pediatr Res.

[24] Morowitz MJ, Poroyko V, Caplan M, Alverdy J, Liu DC (2010). Redefining the role of intestinal microbes in the pathogenesis of necrotizing enterocolitis. Pediatrics.

[25] Boccia D, Stolfi I, Lana S, Moro ML (2001). Nosocomial necrotising enterocolitis outbreaks: epidemiology and control measures. Eur J Pediatr.

[26] Grave GD, Nelson SA, Walker WA, Moss RL, Dvorak B, Hamilton FA, Higgins R, Raju TN (2007). New therapies and preventive approaches for necrotizing enterocolitis: report of a research planning workshop. Pediatr Res.

[27] Raveh-Sadka T, Thomas BC, Singh A, Firek B, Brooks B, Castelle CJ, Sharon I, Baker R, Good M, Morowitz MJ, Banfield JF. Gut bacteria are rarely shared by co-hospitalized premature infants, regardless of necrotizing enterocolitis development. Elife. 2015;4:e05477. 10.7554/eLife.05477 PMC4384745 25735037

[28] Erickson AR, Cantarel BL, Lamendella R, Darzi Y, Mongodin EF, Pan C, Shah M, Halfvarson J, Tysk C, Henrissat B (2012). Integrated metagenomics/metaproteomics reveals human host-microbiota signatures of Crohn’s disease. PLoS One.

[29] Young JC, Pan C, Adams RM, Brooks B, Banfield JF, Morowitz MJ, Hettich RL (2015). Metaproteomics reveals functional shifts in microbial and human proteins during a preterm infant gut colonization case. Proteomics.

[30] Xiong W, Giannone RJ, Morowitz MJ, Banfield JF, Hettich RL (2015). Development of an enhanced metaproteomic approach for deepening the microbiome characterization of the human infant gut. J Proteome Res.

[31] Raveh-Sadka T, Firek B, Sharon I, Baker R, Brown CT, Thomas BC, Morowitz MJ, Banfield JF (2016). Evidence for persistent and shared bacterial strains against a background of largely unique gut colonization in hospitalized premature infants. ISME J.

[32] Giannone RJ, Wurch LL, Heimerl T, Martin S, Yang Z, Huber H, Rachel R, Hettich RL, Podar M (2015). Life on the edge: functional genomic response of Ignicoccus hospitalis to the presence of Nanoarchaeum equitans. ISME J.

[33] Poudel S, Giannone RJ, Rodriguez M, Raman B, Martin MZ, Engle NL, Mielenz JR, Nookaew I, Brown SD, Tschaplinski TJ (2017). Integrated omics analyses reveal the details of metabolic adaptation of Clostridium thermocellum to lignocellulose-derived growth inhibitors released during the deconstruction of switchgrass. Biotechnol Biofuels.

[34] David LA, Maurice CF, Carmody RN, Gootenberg DB, Button JE, Wolfe BE, Ling AV, Devlin AS, Varma Y, Fischbach MA (2014). Diet rapidly and reproducibly alters the human gut microbiome. Nature.

[35] Huerta-Cepas J, Szklarczyk D, Forslund K, Cook H, Heller D, Walter MC, Rattei T, Mende DR, Sunagawa S, Kuhn M (2016). eggNOG 4.5: a hierarchical orthology framework with improved functional annotations for eukaryotic, prokaryotic and viral sequences. Nucleic Acids Res.

[36] Gritz EC, Bhandari V (2015). The human neonatal gut microbiome: a brief review. Front Pediatr.

[37] Jia B, Raphenya AR, Alcock B, Waglechner N, Guo P, Tsang KK, Lago BA, Dave BM, Pereira S, Sharma AN (2017). CARD 2017: expansion and model-centric curation of the comprehensive antibiotic resistance database. Nucleic Acids Res.

[38] Kolmeder CA, Salojarvi J, Ritari J, de Been M, Raes J, Falony G, Vieira-Silva S, Kekkonen RA, Corthals GL, Palva A (2016). Faecal metaproteomic analysis reveals a personalized and stable functional microbiome and limited effects of a probiotic intervention in adults. PLoS One.

[39] Darzi Y, Falony G, Vieira-Silva S, Raes J (2016). Towards biome-specific analysis of meta-omics data. ISME J.

[40] Mu H, Hoy CE (2004). The digestion of dietary triacylglycerols. Prog Lipid Res.

[41] Feehily C, Karatzas KA (2013). Role of glutamate metabolism in bacterial responses towards acid and other stresses. J Appl Microbiol.

[42] Ballard O, Morrow AL (2013). Human milk composition: nutrients and bioactive factors. Pediatr Clin North Am.

[43] Coyne MJ, Reinap B, Lee MM, Comstock LE (2005). Human symbionts use a host-like pathway for surface fucosylation. Science.

[44] Bode L (2009). Human milk oligosaccharides: prebiotics and beyond. Nutr Rev.

[45] Underwood MA, German JB, Lebrilla CB, Mills DA (2015). Bifidobacterium longum subspecies infantis: champion colonizer of the infant gut. Pediatr Res.

[46] Washburn MP, Wolters D, Yates JR (2001). Large-scale analysis of the yeast proteome by multidimensional protein identification technology. Nat Biotechnol.

[47] Abraham P, Adams R, Giannone RJ, Kalluri U, Ranjan P, Erickson B, Shah M, Tuskan GA, Hettich RL (2012). Defining the boundaries and characterizing the landscape of functional genome expression in vascular tissues of Populus using shotgun proteomics. J Proteome Res.

[48] Robinson MD, McCarthy DJ (2010). Smyth GK: edgeR: a Bioconductor package for differential expression analysis of digital gene expression data. Bioinformatics.

[49] Moriya Y, Itoh M, Okuda S, Yoshizawa AC, Kanehisa M (2007). KAAS: an automatic genome annotation and pathway reconstruction server. Nucleic Acids Res.

[50] Vieira-Silva S, Falony G, Darzi Y, Lima-Mendez G, Garcia Yunta R, Okuda S, Vandeputte D, Valles-Colomer M, Hildebrand F, Chaffron S, Raes J (2016). Species-function relationships shape ecological properties of the human gut microbiome. Nat Microbiol.

