# Supplementary figures and images for: Genome-resolved metaproteomic characterization of preterm infant gut microbiota development reveals species-specific metabolic shifts and variabilities during early life
Source: Microbiome. 2017 Jul 10;5:72. doi: 10.1186/s40168-017-0290-6 (PMC5504695; doi:10.1186/s40168-017-0290-6)

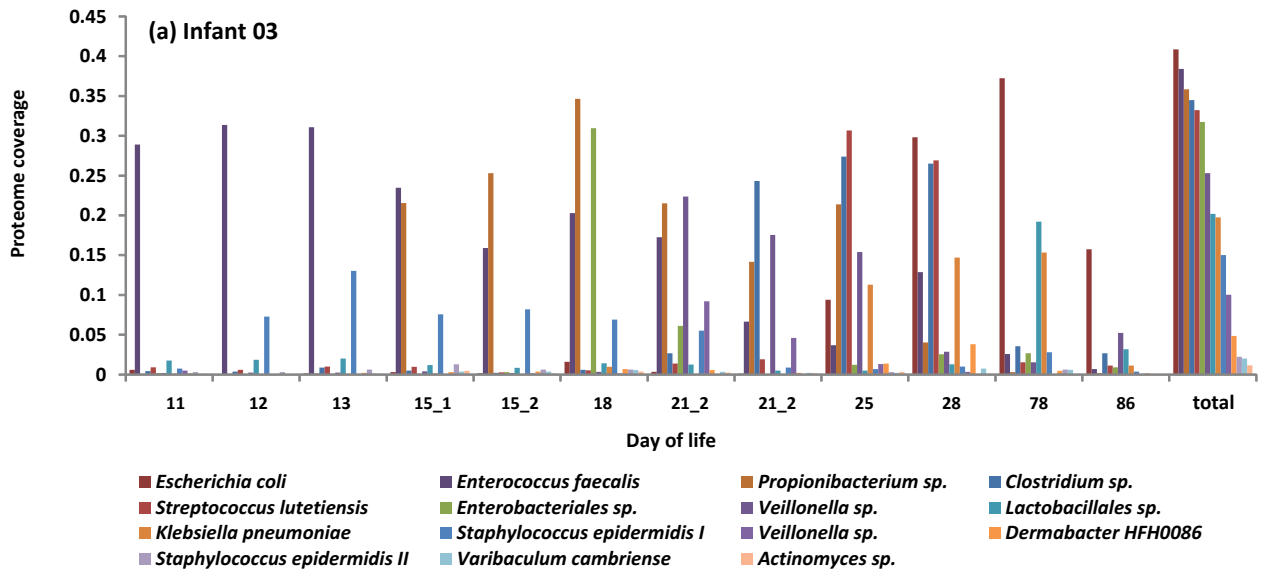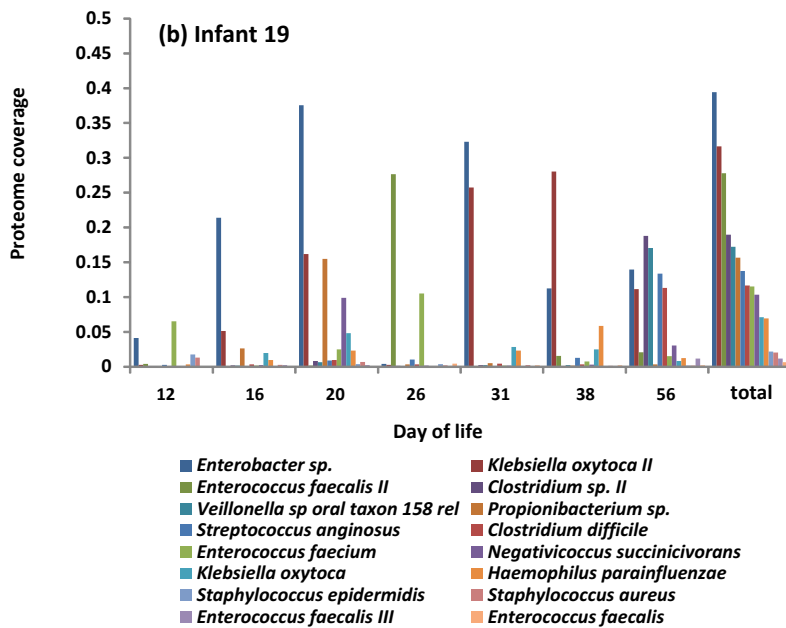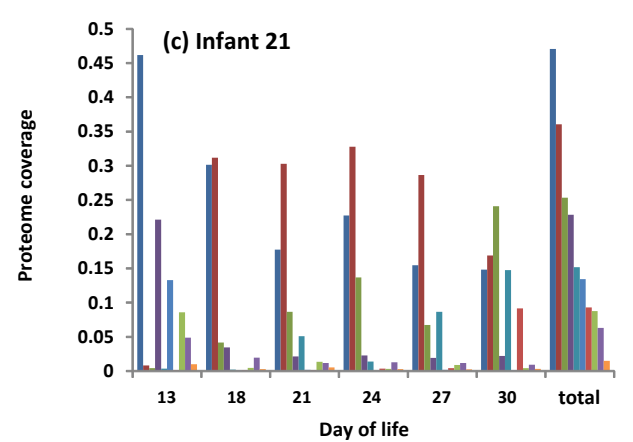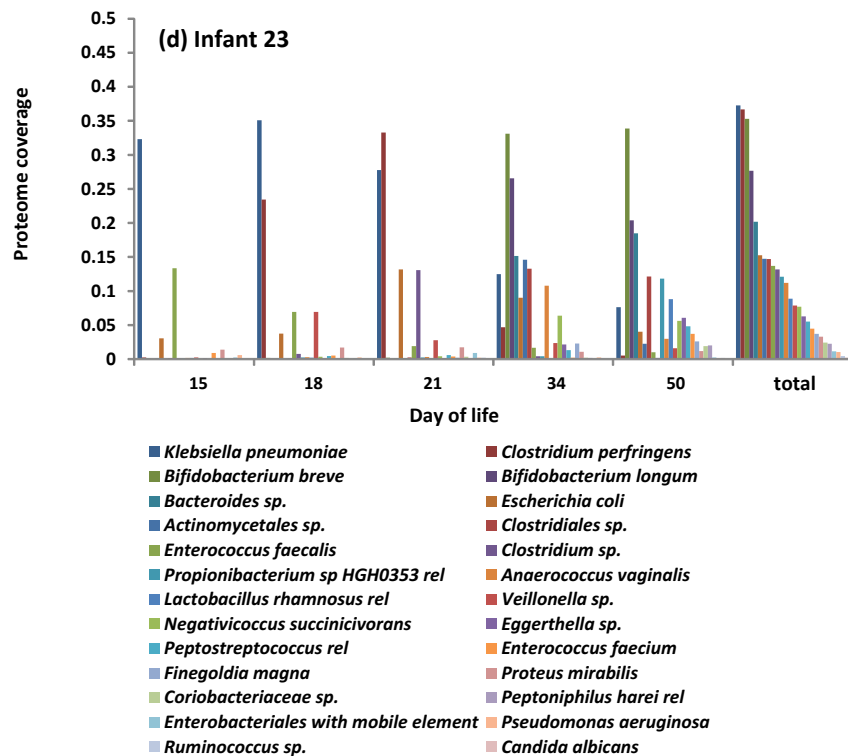

Supplement: Supplementary file 3 — Organism-specific proteome coverage across time series of (a) infant 03, (b) infant 19, (c) infant 21, and (d) infant 23. The percentage of proteome identified is calculated for each organism by assigning peptides to proteins predicted from metagenomics data. Sample with day of life (DOL) is shown on the x-axis, and “total” column includes proteins identified across time series. (PDF 39 kb) [file 40168_2017_290_MOESM3_ESM.pdf]

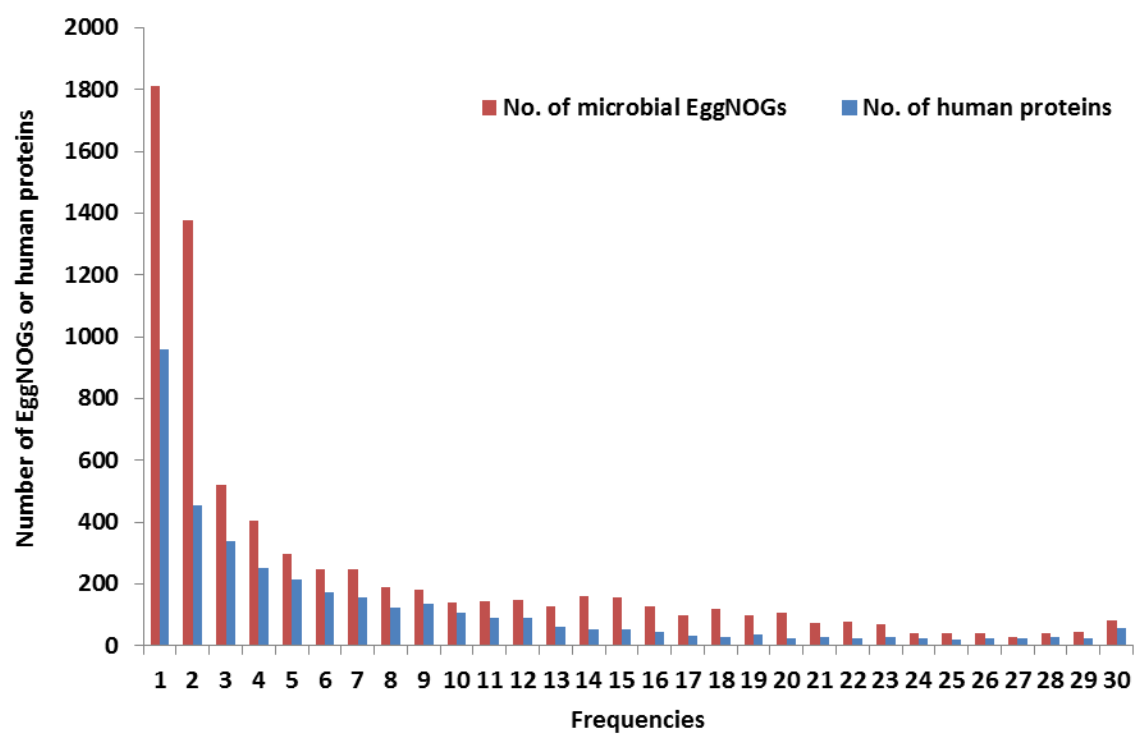

Supplement: Supplementary file 4 — Frequencies of microbial EggNOGs and human protein groups identified across all samples. 81 microbial EggNOGs and 57 human protein groups are identified in all 30 samples. (PDF 92 kb) [file 40168_2017_290_MOESM4_ESM.pdf]

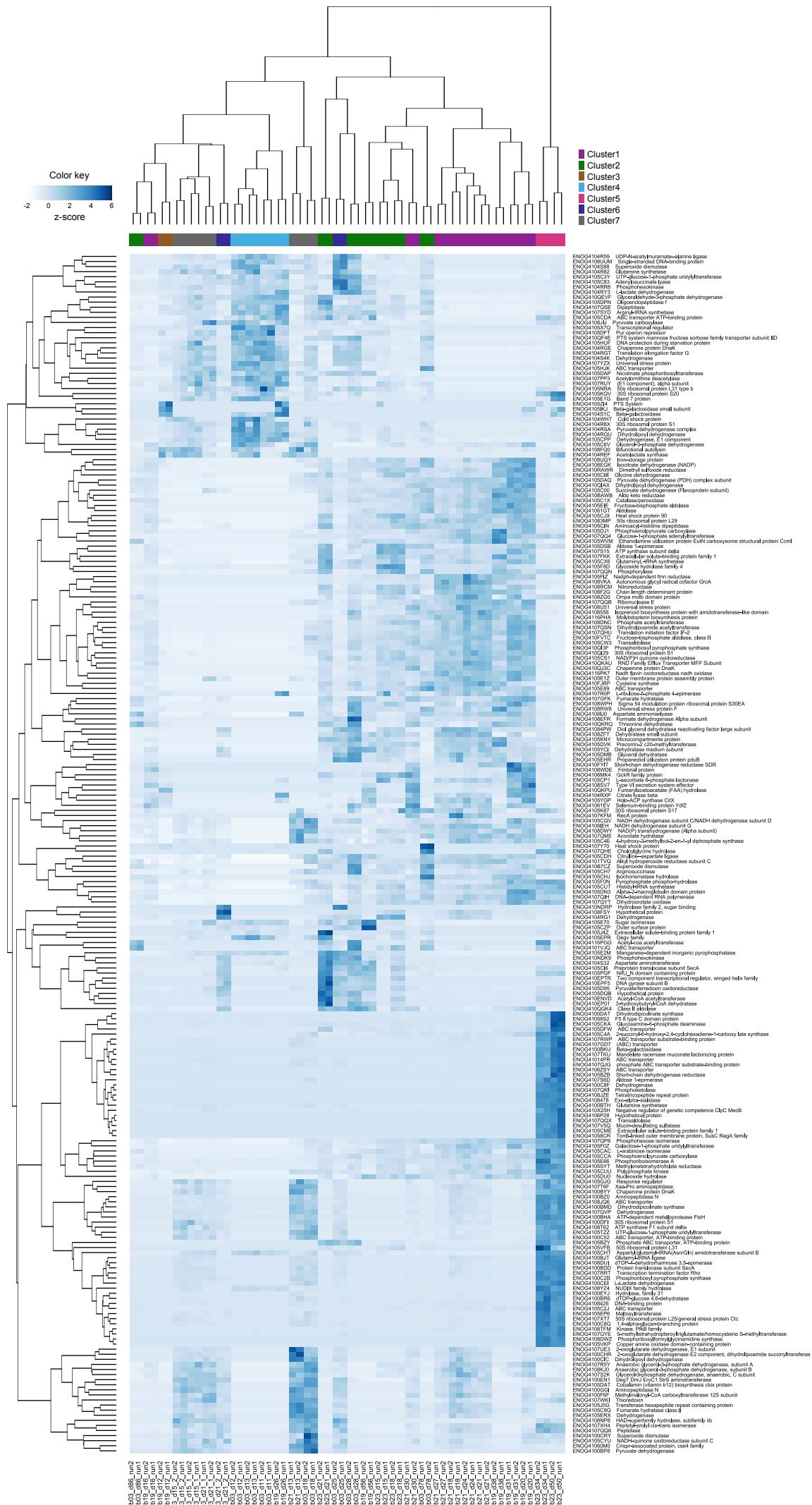

Supplement: Supplementary file 7 — Proteins associated with metaproteome clusters. Shown are top 25 most significantly different protein groups between each metaproteome cluster pair comparison with p-value less than 0.01. Metaproteome clusters are indicated by colored boxes on top of the heatmap. Sample names at the bottom are composed of the infant number (b), day of life (d) and the measurement number (run). (PDF 2441 kb) [file 40168_2017_290_MOESM7_ESM.pdf]

## Propionate

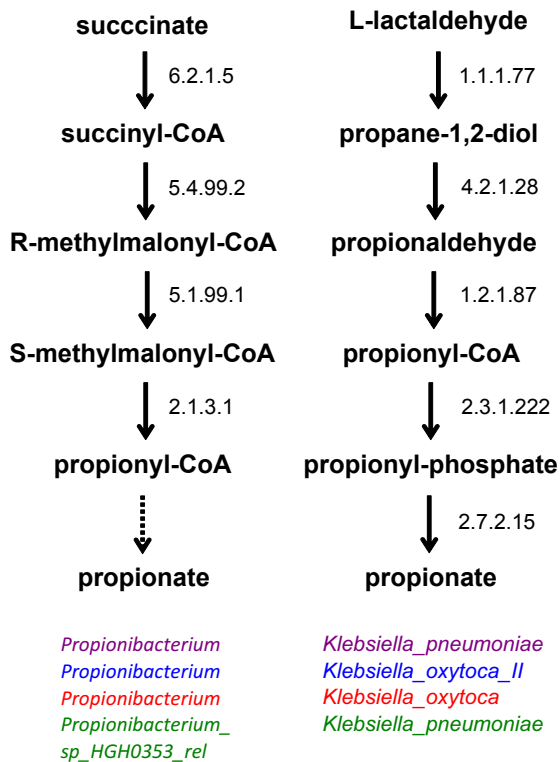

## Butyrate

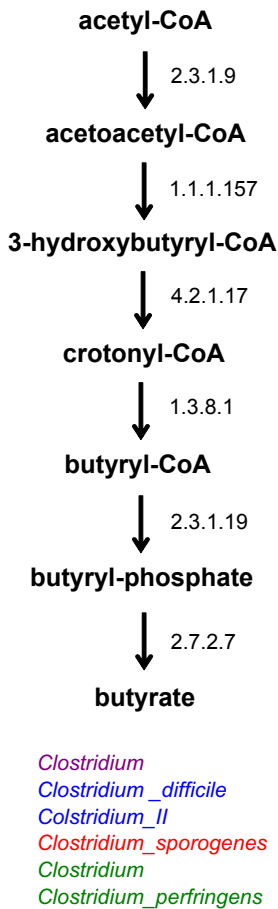

## Acetate

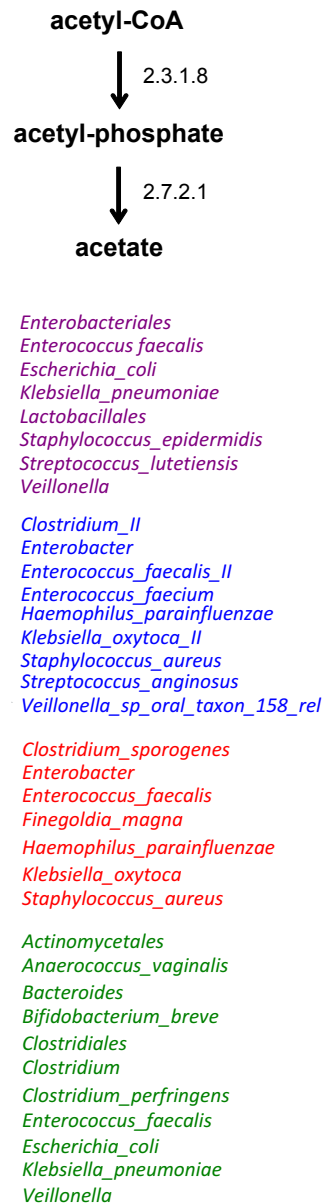

- Infant 03
- Infant 19
- Infant 21
- Infant 23

Supplement: Supplementary file 9 — Metabolic pathways for propionate, butyrate and acetate formation by representative bacterial species/strains in human preterm infant gut. Species/strains with all enzymes (shown by EC number) in the pathway identified are listed below. The color indicates in which infant the species/strain is identified. (6.2.1.5: succinyl-CoA sythetase; 5.4.99.2: methylmalonyl-CoA mutase; 5.1.99.1: methylmalonyl-CoA epimerase; 2.1.3.1: methylmalonyl-CoA carboxyltransferase; 1.1.1.77: lactaldehyde reductase; 4.2.1.28: propanediol dehydratase; 1.2.1.87: propionaldehyde dehydrogenase; 2.3.1.222: phosphate propanoyltransferase; 2.7.2.15: propionate kinase; 2.3.1.9: acetyl-CoA C-acetyltransferase; 1.1.1.157: 3-hydroxybutyryl-CoA dehydrogenase; 4.2.1.17: enoyl-CoA hydratase; 1.3.8.1: crotonyl-CoA reductase; 2.3.1.19: phosphotransbutyrylase; 2.7.2.7: butyrate kinase; 2.3.1.8: phosphate acetyltransferase; 2.7.2.1: acetate kinase). (PDF 44 kb) [file 40168_2017_290_MOESM9_ESM.pdf]
